# Supplementary material for: Fatty Acids of European Sardine (Sardina pilchardus) White Muscle Can Discriminate Geographic Origin Along the Iberian Atlantic Coast
Source: Foods. 2025 Jan 3;14(1):120. doi: 10.3390/foods14010120 (PMC11720401; doi:10.3390/foods14010120)
Supplement: Supplementary file 1 [file foods-14-00120-s001.zip › foods-3341218-supplementary.pdf]

Supplementary Table S1

Average fatty acid (FA) profiles (relative abundance of total pool of FA, %, ± SD) of *Sardina pilchardus* white muscle, sampled from individuals obtained in the seven landing locations along the Iberian Atlantic coast (n = 10 per location). Cor: Coruña; RP: Ria de Pontevedra; VC: Viana do Castelo; Mat: Matosinhos; Pe: Peniche; Ses: Sesimbra; Por: Portimão.

**Fatty Acid**	**Cor1**	**Cor2**	**Cor3**	**Cor4**	**Cor5**	**Cor6**	**Cor7**	**Cor8**	**Cor9**	**Cor10**
**C12:0**										
**C14:0**	2.997	3.756	3.151	3.340	3.923	3.696	3.735	3.342	4.467	3.308
**13-methyl-tetradecanoic acid**	0.000	0.000	0.000	0.000	0.000	0.000	0.000	0.000	0.000	0.000
**C15:0**	0.405	0.528	0.466	0.347	0.485	0.424	0.477	0.360	0.415	0.424
**13-methyl-pentadecanoic acid**	0.000	0.000	0.000	0.000	0.000	0.000	0.000	0.000	0.000	0.000
**C16:0**	19.238	19.083	26.185	17.354	20.234	16.983	18.588	19.378	21.200	17.172
**C16:1*n*-7**	3.824	4.031	3.822	5.777	4.225	4.205	3.910	4.465	5.834	3.753
**15-methyl-hexadecanoic acid**	0.000	0.000	0.000	0.000	0.000	0.000	0.000	0.000	0.000	0.000
**C16:2*n*-4**	0.277	0.321	0.000	0.580	0.339	0.339	0.355	0.420	0.580	0.318
**7-methyl-6-hexadecenoic acid**	0.379	0.476	0.000	0.231	0.000	0.376	0.344	0.271	0.000	0.400
**C17:0**	0.000	0.000	0.000	0.000	0.000	0.000	0.000	0.000	0.000	0.000
**C16:3*n*-4**	0.168	0.122	0.000	0.383	0.221	0.200	0.210	0.288	0.392	0.186
**C17:1*n*-9**	0.000	0.000	0.000	0.000	0.000	0.000	0.000	0.000	0.000	0.000
**C16:4*n*-1**	0.323	0.271	0.000	1.103	0.404	0.459	0.483	0.636	0.874	0.363
**C18:0**	7.281	5.878	10.840	5.086	7.265	4.841	5.360	6.036	6.863	4.567
**C18:1*n*-9**	10.879	10.882	6.353	5.539	7.808	11.395	8.935	7.075	5.153	9.099
**C18:1*n*-6**	2.244	2.012	2.092	2.276	2.014	1.869	1.944	2.046	2.242	1.933
**C18:1*n*-5**	0.000	0.000	0.000	0.000	0.000	0.000	0.000	0.000	0.000	0.000
**C18:2*n*-6**	1.034	1.054	0.000	0.670	0.698	1.245	1.129	0.654	0.768	0.939
**C18:2*n*-5**	0.000	0.000	0.000	0.000	0.000	0.000	0.000	0.000	0.000	0.000
**C18:3*n*-3**	0.741	0.912	0.000	0.471	0.000	1.073	0.856	0.467	0.483	0.854
**C18:4*n*-3**	2.124	2.434	2.082	2.033	1.672	3.034	2.368	1.829	2.071	2.465
**C18:4*n*-1**	0.000	0.000	0.000	0.000	0.000	0.000	0.000	0.000	0.000	0.000
**C20:0**	0.000	0.000	0.000	0.000	0.000	0.000	0.000	0.000	0.000	0.000
**C20:1*n*-11**	3.073	3.972	2.668	2.665	3.746	3.951	3.937	2.967	1.946	4.410
**C20:1*n*-9**	0.000	0.000	0.000	0.000	0.000	0.000	0.000	0.000	0.000	0.000
**Δ 5.13 20:2**	0.000	0.000	0.000	0.000	0.000	0.000	0.000	0.000	0.000	0.000
**C20:4*n*-6**	0.557	0.740	0.491	0.597	0.656	0.704	0.639	0.746	0.494	0.663
**C20:4*n*-3**	0.000	0.797	0.000	0.632	0.479	1.052	0.772	0.508	0.432	0.695
**C20:5*n*-3**	11.740	12.088	11.704	20.204	11.501	12.332	12.077	14.895	18.496	12.507
**C22:1*n*-11**	5.500	6.255	3.406	4.939	7.186	6.642	7.090	5.872	4.479	8.162
**C22:4*n*-6**	0.187	0.316	0.000	0.438	0.000	0.385	0.295	0.299	0.000	0.302
**C22:5*n*-6**	0.000	0.000	0.000	0.000	0.000	0.000	0.000	0.000	0.000	0.000
**C22:5*n*-*3***	0.919	1.489	1.273	1.853	1.110	1.430	1.474	1.611	0.961	1.278
**C22:6*n*-3**	26.110	22.582	25.468	23.483	26.035	23.363	25.021	25.837	21.851	26.202
**∑SFA**	29.921	29.245	40.642	26.127	31.907	25.944	28.160	29.116	32.944	25.471
**∑MUFA**	25.521	27.152	18.341	21.196	24.979	28.063	25.816	22.424	19.654	27.357
**∑PUFA**	44.180	43.127	41.017	52.446	43.114	45.616	45.680	48.188	47.401	46.773
**∑*n*-3**	41.634	40.303	40.526	48.675	40.796	42.284	42.569	45.147	44.293	44.001

Supplementary Table S1 (cont.)

Fatty Acid	RP1	RP2	RP3	RP4	RP5	RP6	RP7	RP8	RP9	RP10
**C12:0**										
**C14:0**	4.725	4.431	2.801	3.690	3.247	3.074	5.275	5.604	2.865	5.071
**13-methyl-tetradecanoic acid**	0.000	0.000	0.000	0.000	0.000	0.000	0.000	0.000	0.000	0.000
**C15:0**	0.506	0.594	0.493	0.369	0.430	0.363	0.393	0.686	0.711	0.355
**13-methyl-pentadecanoic acid**	0.000	0.000	0.000	0.000	0.000	0.000	0.000	0.000	0.000	0.000
**C16:0**	23.900	27.554	22.760	20.604	21.878	20.111	20.864	27.723	24.483	23.198
**C16:1*n*-7**	7.212	7.627	4.497	7.693	5.919	6.372	9.343	9.545	6.347	8.943
**15-methyl-hexadecanoic acid**	0.000	0.000	0.000	0.000	0.000	0.000	0.000	0.000	0.000	0.000
**C16:2*n*-4**	0.000	0.000	0.000	0.000	0.000	0.000	0.000	0.000	0.000	0.000
**7-methyl-6-hexadecenoic acid**	0.000	0.000	0.000	0.000	0.000	0.000	0.000	0.000	0.000	0.000
**C17:0**	0.000	0.000	0.000	0.000	0.000	0.000	0.000	0.000	0.000	0.000
**C16:3*n*-4**	0.000	0.000	0.000	0.000	0.000	0.000	0.000	0.000	0.000	0.000
**C17:1*n*-9**	0.000	0.000	0.000	0.000	0.000	0.000	0.000	0.000	0.000	0.000
**C16:4*n*-1**	0.724	0.801	0.000	1.265	0.611	0.908	1.810	0.000	0.000	1.401
**C18:0**	13.106	10.446	12.177	8.775	9.211	7.258	7.506	8.240	14.637	8.248
**C18:1*n*-9**	5.729	8.592	3.662	7.072	7.026	9.294	9.129	6.122	4.238	10.843
**C18:1*n*-6**	2.835	3.177	2.770	3.159	2.915	2.910	3.511	3.180	2.425	3.170
**C18:1*n*-5**	0.000	0.000	0.000	0.000	0.000	0.000	0.000	0.000	0.000	0.000
**C18:2*n*-6**	0.000	0.000	0.000	0.399	0.590	0.559	0.503	0.000	0.000	0.455
**C18:2*n*-5**	0.000	0.000	0.000	0.000	0.000	0.000	0.000	0.000	0.000	0.000
**C18:3*n*-3**	0.000	0.000	0.000	0.000	0.000	0.000	0.000	0.000	0.000	0.000
**C18:4*n*-3**	2.050	1.644	1.708	2.017	2.300	1.937	1.855	2.500	1.170	1.566
**C18:4*n*-1**	0.000	0.000	0.000	0.000	0.000	0.000	0.000	0.000	0.000	0.000
**C20:0**	0.000	0.000	0.000	0.000	0.000	0.000	0.000	0.000	0.000	0.000
**C20:1*n*-11**	0.000	0.000	0.000	0.000	0.000	0.000	0.000	0.000	0.000	0.000
**C20:1*n*-9**	0.000	0.000	0.000	0.000	0.000	0.000	0.000	0.000	0.000	0.000
**Δ 5.13 20:2**	0.000	0.000	0.000	0.000	0.000	0.000	0.000	0.000	0.000	0.000
**C20:4*n*-6**	0.000	0.000	0.000	0.000	0.000	0.000	0.000	0.000	0.000	0.000
**C20:4*n*-3**	0.000	0.000	0.000	0.000	0.000	0.000	0.000	0.000	0.000	0.000
**C20:5*n*-3**	17.530	18.218	13.121	25.052	19.214	23.456	25.678	17.478	11.730	22.391
**C22:1*n*-11**	0.000	0.000	0.000	0.000	0.000	0.000	0.000	0.000	0.000	0.000
**C22:4*n*-6**	0.000	0.000	0.000	0.000	0.000	0.000	0.000	0.000	0.000	0.000
**C22:5*n*-6**	0.000	0.000	0.000	0.000	0.000	0.000	0.000	0.000	0.000	0.000
**C22:5*n-3***	0.832	0.738	0.792	1.193	1.133	1.705	1.862	1.931	1.601	1.636
**C22:6*n*-3**	20.850	16.179	35.220	18.710	25.526	22.052	12.273	16.990	29.793	12.723
**∑SFA**	42.237	43.024	38.231	33.439	34.766	30.807	34.036	42.253	42.696	36.872
**∑MUFA**	15.776	19.396	10.929	17.925	15.860	18.576	21.984	18.847	13.010	22.956
**∑PUFA**	41.987	37.580	50.840	48.636	49.374	50.617	43.980	38.900	44.294	40.173
**∑*n*-3**	41.263	36.779	50.840	46.972	48.173	49.150	41.667	38.900	44.294	38.316

Supplementary Table S1 (cont.)

**Fatty Acid**	**VC1**	**VC2**	**VC3**	**VC4**	**VC5**	**VC6**	**VC7**	**VC8**	**VC9**	**VC10**
**C12:0**										
**C14:0**	3.773	4.179	3.042	4.059	4.117	4.040	3.798	3.974	3.699	3.605
**13-methyl-tetradecanoic acid**	0.078	0.068	0.079	0.000	0.045	0.000	0.000	0.090	0.000	0.078
**C15:0**	0.332	0.283	0.334	0.320	0.355	0.401	0.368	0.334	0.393	0.377
**13-methyl-pentadecanoic acid**	0.000	0.000	0.000	0.000	0.000	0.000	0.000	0.000	0.000	0.000
**C16:0**	17.326	15.994	21.613	17.923	17.039	19.759	15.776	13.631	16.661	18.386
**C16:1*n*-7**	6.350	7.771	5.104	5.700	6.778	5.858	5.627	5.575	5.664	5.127
**15-methyl-hexadecanoic acid**	0.197	0.213	0.000	0.112	0.145	0.000	0.000	0.160	0.043	0.147
**C16:2*n*-4**	0.540	0.756	0.000	0.000	0.524	0.000	0.000	0.616	0.000	0.399
**7-methyl-6-hexadecenoic acid**	0.000	0.000	0.000	0.000	0.000	0.000	0.000	0.000	0.000	0.000
**C17:0**	0.000	0.000	0.000	0.000	0.000	0.000	0.000	0.000	0.000	0.000
**C16:3*n*-4**	0.000	0.000	0.000	0.000	0.000	0.000	0.000	0.000	0.000	0.000
**C17:1*n*-9**	0.000	0.000	0.000	0.000	0.000	0.000	0.000	0.000	0.000	0.000
**C16:4*n*-1**	1.038	2.127	0.000	0.672	0.881	0.000	0.000	0.875	0.000	0.579
**C18:0**	6.227	5.092	7.571	5.279	5.105	6.318	5.212	4.659	5.677	5.712
**C18:1*n*-9**	8.640	9.162	12.493	8.403	7.609	6.829	10.455	8.001	9.412	8.198
**C18:1*n*-6**	2.399	2.858	2.163	2.508	2.569	2.429	2.663	2.462	2.577	2.107
**C18:1*n*-5**	0.000	0.000	0.000	0.000	0.000	0.000	0.000	0.000	0.000	0.000
**C18:2*n*-6**	0.819	0.593	0.592	0.689	0.953	0.581	1.056	0.773	0.812	0.950
**C18:2*n*-5**	0.000	0.000	0.000	0.000	0.000	0.000	0.000	0.000	0.000	0.000
**C18:3*n*-3**	0.624	0.375	0.345	0.468	0.582	0.380	0.884	0.532	0.630	0.824
**C18:4*n*-3**	2.406	2.293	1.442	1.925	2.334	1.889	2.581	2.210	1.960	2.232
**C18:4*n*-1**	0.000	0.000	0.000	0.000	0.000	0.000	0.000	0.000	0.000	0.000
**C20:0**	0.000	0.000	0.000	0.000	0.000	0.000	0.000	0.000	0.000	0.000
**C20:1*n*-11**	2.286	2.076	2.409	3.899	2.693	2.632	3.527	5.759	5.428	4.514
**C20:1*n*-9**	0.000	0.000	0.000	0.000	0.000	0.000	0.000	0.000	0.000	0.000
**Δ 5.13 20:2**	0.000	0.000	0.000	0.000	0.000	0.000	0.000	0.000	0.000	0.000
**C20:4*n*-6**	0.590	0.530	0.000	0.000	0.000	0.000	1.379	1.109	0.000	0.783
**C20:4*n*-3**	0.657	0.581	0.000	0.000	0.000	0.000	0.932	0.790	0.000	0.757
**C20:5*n*-3**	20.017	24.848	12.348	15.719	19.319	16.822	16.662	16.326	16.087	14.061
**C22:1*n*-11**	2.935	1.963	3.019	7.063	3.683	3.933	5.816	9.832	9.032	8.585
**C22:4*n*-6**	0.000	0.000	0.000	0.000	0.000	0.000	0.000	0.000	0.000	0.000
**C22:5*n*-6**	0.000	0.000	0.000	0.000	0.000	0.000	0.000	0.000	0.000	0.000
**C22:5*n-3***	1.538	2.296	1.626	1.680	1.697	1.846	2.042	2.406	2.179	1.762
**C22:6*n*-3**	21.230	15.942	25.821	23.582	23.570	26.284	21.223	19.885	19.748	20.818
**∑SFA**	27.659	25.548	32.561	27.581	26.616	30.518	25.153	22.598	26.430	28.079
**∑MUFA**	22.609	23.829	25.187	27.573	23.333	21.680	28.088	31.628	32.112	28.532
**∑PUFA**	49.457	50.341	42.173	44.734	49.860	47.802	46.759	45.524	41.415	43.164
**∑*n*-3**	46.471	46.335	41.581	43.373	47.502	47.221	44.324	42.151	40.603	40.454

Supplementary Table S1 (cont.)

Fatty acid	Mat1	Mat2	Mat3	Mat4	Mat5	Mat6	Mat7	Mat8	Mat9	Mat10
**C12:0**										
**C14:0**	2.721	2.999	3.838	3.726	3.472	3.474	4.207	5.578	4.170	3.417
**13-methyl-tetradecanoic acid**	0.000	0.091	0.098	0.000	0.000	0.097	0.105	0.127	0.107	0.100
**C15:0**	0.355	0.311	0.370	0.348	0.396	0.282	0.328	0.409	0.414	0.325
**13-methyl-pentadecanoic acid**	0.000	0.000	0.000	0.000	0.000	0.000	0.000	0.000	0.000	0.000
**C16:0**	17.658	15.562	16.404	15.133	15.870	15.744	17.136	21.740	18.942	17.265
**C16:1*n*-7**	4.016	4.966	5.714	5.138	5.660	4.785	4.824	7.002	5.475	4.755
**15-methyl-hexadecanoic acid**	0.000	0.166	0.244	0.000	0.000	0.158	0.143	0.162	0.203	0.184
**C16:2*n*-4**	0.000	0.399	0.469	0.465	0.458	0.437	0.485	0.537	0.481	0.415
**7-methyl-6-hexadecenoic acid**	0.000	0.000	0.000	0.000	0.000	0.000	0.000	0.000	0.000	0.000
**C17:0**	0.000	0.324	0.000	0.130	0.000	0.000	0.131	0.133	0.163	0.129
**C16:3*n*-4**	0.000	0.307	0.358	0.384	0.418	0.313	0.346	0.418	0.387	0.263
**C17:1*n*-9**	0.000	0.000	0.000	0.000	0.000	0.000	0.000	0.000	0.000	0.000
**C16:4*n*-1**	0.000	0.586	0.748	0.698	0.728	0.606	0.560	0.785	0.772	0.589
**C18:0**	8.865	5.159	5.718	4.763	5.625	4.466	4.886	4.916	5.717	4.986
**C18:1*n*-9**	6.532	8.459	9.518	9.048	5.666	6.730	7.350	6.982	6.459	8.600
**C18:1*n*-6**	2.151	2.254	2.376	2.387	2.528	2.265	2.271	2.362	2.338	2.319
**C18:1*n*-5**	0.000	0.000	0.000	0.000	0.000	0.000	0.000	0.000	0.000	0.000
**C18:2*n*-6**	0.702	0.746	0.740	0.883	0.589	0.700	0.899	0.622	0.708	0.705
**C18:2*n*-5**	0.000	0.000	0.000	0.000	0.000	0.000	0.000	0.000	0.000	0.000
**C18:3*n*-3**	0.000	0.514	0.527	0.753	0.442	0.490	0.684	0.480	0.521	0.546
**C18:4*n*-3**	1.609	2.236	2.121	2.707	1.968	1.926	2.237	2.320	2.156	2.001
**C18:4*n*-1**	0.000	0.000	0.000	0.000	0.000	0.000	0.000	0.000	0.000	0.000
**C20:0**	0.000	0.000	0.153	0.226	0.000	0.073	0.144	0.000	0.119	0.096
**C20:1*n*-11**	2.904	2.771	2.896	4.135	2.456	3.683	5.088	2.950	2.713	3.393
**C20:1*n*-9**	0.000	0.000	0.118	0.000	0.000	0.097	0.098	0.000	0.096	0.064
**Δ 5.13 20:2**	0.000	0.000	0.000	0.000	0.000	0.000	0.000	0.000	0.000	0.000
**C20:4*n*-6**	1.087	0.886	1.037	0.980	1.147	1.034	0.935	0.000	0.655	1.054
**C20:4*n*-3**	0.469	0.592	0.626	0.964	0.000	0.531	0.000	0.446	0.591	0.599
**C20:5*n*-3**	13.258	17.954	16.610	14.881	17.952	15.910	13.902	14.467	17.126	15.941
**C22:1*n*-11**	5.364	5.539	4.751	7.791	5.190	7.410	8.775	5.674	4.977	6.002
**C22:4*n*-6**	0.000	0.411	0.439	0.506	0.443	0.000	0.375	0.000	0.000	0.384
**C22:5*n*-6**	0.000	0.000	0.000	0.000	0.000	0.000	0.000	0.000	0.000	0.000
**C22:5*n-3***	1.566	1.984	2.020	2.124	2.060	1.944	1.475	2.559	1.510	1.764
**C22:6*n*-3**	30.742	24.786	22.104	21.829	26.932	26.846	22.615	19.327	23.199	24.106
**∑SFA**	29.599	24.354	26.484	24.325	25.363	24.039	26.833	32.776	29.526	26.218
**∑MUFA**	20.967	23.988	25.374	28.499	21.500	24.970	28.406	24.970	22.059	25.132
**∑PUFA**	49.434	51.400	47.799	47.175	53.137	50.736	44.514	41.963	48.105	48.366
**∑*n*-3**	47.644	48.066	44.008	43.259	49.354	47.646	40.914	39.600	45.103	44.957

Supplementary Table S1 (cont.)

Fatty acid	Pe1	Pe2	Pe3	Pe4	Pe5	Pe6	Pe7	Pe8	Pe9	Pe10
**C12:0**										
**C14:0**	3.546	3.590	3.855	2.914	3.333	3.671	3.227	3.280	3.787	3.153
**13-methyl-tetradecanoic acid**	0.076	0.053	0.058	0.000	0.000	0.073	0.000	0.000	0.046	0.020
**C15:0**	0.281	0.281	0.251	0.371	0.261	0.365	0.302	0.282	0.270	0.278
**13-methyl-pentadecanoic acid**	0.000	0.000	0.000	0.000	0.000	0.000	0.000	0.000	0.000	0.000
**C16:0**	14.839	15.066	15.436	15.681	20.476	17.185	17.624	17.286	18.206	18.362
**C16:1*n*-7**	7.002	7.148	5.930	5.403	5.971	6.579	5.973	7.011	6.634	6.311
**15-methyl-hexadecanoic acid**	0.227	0.234	0.242	0.186	0.000	0.282	0.193	0.000	0.231	0.213
**C16:2*n*-4**	0.602	0.639	0.602	0.349	0.000	0.601	0.516	0.670	0.632	0.561
**7-methyl-6-hexadecenoic acid**	0.174	0.171	0.162	0.305	0.000	0.216	0.174	0.092	0.101	0.000
**C17:0**	0.114	0.111	0.112	0.177	0.000	0.207	0.104	0.000	0.000	0.106
**C16:3*n*-4**	0.680	0.729	0.590	0.312	0.000	0.683	0.535	0.700	0.646	0.584
**C17:1*n*-9**	0.000	0.000	0.000	0.000	0.000	0.000	0.000	0.000	0.000	0.000
**C16:4*n*-1**	1.974	2.002	1.608	0.966	1.440	2.092	1.438	2.061	1.891	1.769
**C18:0**	5.678	5.926	6.649	6.398	8.984	6.263	8.551	7.533	8.421	8.250
**C18:1*n*-9**	8.623	10.307	10.111	8.992	9.921	8.323	9.080	7.621	8.234	8.093
**C18:1*n*-6**	3.245	3.241	2.877	2.918	2.910	3.152	3.173	3.567	3.245	3.281
**C18:1*n*-5**	0.000	0.000	0.000	0.000	0.000	0.000	0.000	0.000	0.000	0.000
**C18:2*n*-6**	0.642	0.563	0.497	0.894	0.479	0.660	0.659	0.524	0.502	0.469
**C18:2*n*-5**	0.258	0.313	0.280	0.173	0.000	0.277	0.000	0.345	0.232	0.272
**C18:3*n*-3**	0.314	0.290	0.333	0.474	0.000	0.364	0.309	0.272	0.249	0.262
**C18:4*n*-3**	2.124	2.180	1.831	1.787	1.744	2.348	1.880	2.211	1.925	1.988
**C18:4*n*-1**	0.341	0.373	0.377	0.000	0.000	0.297	0.000	0.394	0.287	0.000
**C20:0**	0.147	0.244	0.198	0.215	0.108	0.181	0.237	0.126	0.000	0.081
**C20:1*n*-11**	1.864	2.305	3.058	1.594	1.616	1.542	1.882	1.185	1.700	1.503
**C20:1*n*-9**	0.063	0.108	0.096	0.197	0.000	0.129	0.000	0.000	0.000	0.000
**Δ 5.13 20:2**	0.000	0.000	0.000	0.000	0.000	0.000	0.000	0.000	0.000	0.000
**C20:4*n*-6**	0.677	0.927	1.063	0.705	0.577	0.648	0.685	0.699	0.709	0.677
**C20:4*n*-3**	0.636	0.811	0.763	0.551	0.439	0.634	0.502	0.631	0.482	0.537
**C20:5*n*-3**	24.376	23.305	22.946	19.533	19.572	23.091	20.971	23.578	22.452	22.569
**C22:1*n*-11**	0.000	0.000	0.000	0.000	0.000	0.000	0.000	0.000	0.000	0.000
**C22:4*n*-6**	0.626	0.657	0.639	0.448	0.425	0.670	0.500	0.670	0.464	0.522
**C22:5*n*-6**	0.239	0.176	0.000	0.247	0.138	0.287	0.000	0.210	0.000	0.213
**C22:5*n-3***	2.662	2.797	3.033	2.058	2.015	2.392	2.056	2.815	2.021	2.343
**C22:6*n*-3**	17.970	15.454	16.402	26.154	19.591	16.788	19.428	16.237	16.634	17.585
**∑SFA**	24.605	25.217	26.501	25.756	33.162	27.872	30.045	28.508	30.685	30.230
**∑MUFA**	20.797	23.110	22.073	19.104	20.418	19.725	20.109	19.384	19.813	19.188
**∑PUFA**	54.121	51.215	50.963	54.649	46.420	51.833	49.479	52.016	49.124	50.350
**∑*n*-3**	48.081	44.837	45.308	50.556	43.361	45.617	45.146	45.744	43.763	45.283

Supplementary Table S1 (cont.)

Fatty acid	Ses1	Ses2	Ses3	Ses4	Ses5	Ses6	Ses7	Ses8	Ses9	Ses10
**C12:0**	0.064	0.036	0.064	0.047	0.022	0.03	0	0.041	0	0.039
**C14:0**	3.719	3.038	3.515	4.154	2.627	3.476	3.561	3.340	3.337	3.541
**13-methyl-tetradecanoic acid**	0.115	0.104	0.096	0.121	0.090	0.106	0.104	0.102	0.088	0.105
**C15:0**	0.416	0.328	0.367	0.423	0.327	0.359	0.357	0.367	0.372	0.342
**13-methyl-pentadecanoic acid**	0.094	0.071	0.086	0.090	0.000	0.089	0.086	0.107	0.087	0.090
**C16:0**	14.314	12.656	15.367	15.707	14.041	13.607	13.784	14.939	13.991	13.893
**C16:1*n*-7**	7.154	5.630	5.830	6.114	6.141	5.824	6.854	6.165	6.937	6.349
**15-methyl-hexadecanoic acid**	0.259	0.189	0.203	0.216	0.211	0.200	0.281	0.229	0.258	0.232
**C16:2*n*-4**	0.786	0.529	0.534	0.582	0.459	0.633	0.671	0.615	0.640	0.641
**7-methyl-6-hexadecenoic acid**	0.281	0.344	0.306	0.274	0.268	0.311	0.307	0.271	0.292	0.276
**C17:0**	0.236	0.202	0.159	0.146	0.119	0.149	0.203	0.168	0.169	0.166
**C16:3*n*-4**	0.720	0.423	0.416	0.432	0.395	0.475	0.717	0.517	0.605	0.514
**C17:1*n*-9**	0.133	0.120	0.000	0.000	0.000	0.142	0.108	0.000	0.000	0.132
**C16:4*n*-1**	1.988	1.144	1.308	1.188	1.292	1.184	1.896	1.444	1.781	1.313
**C18:0**	4.621	4.447	5.852	4.618	4.816	4.578	4.647	5.277	4.532	4.675
**C18:1*n*-9**	7.725	8.795	7.869	6.971	5.697	7.364	9.753	9.421	4.883	10.099
**C18:1*n*-6**	2.961	2.687	2.584	2.581	2.802	2.551	2.886	2.694	2.918	2.801
**C18:1*n*-5**	0.035	0.019	0.025	0.000	0.000	0.000	0.040	0.000	0.013	0.051
**C18:2*n*-6**	0.645	0.761	0.710	0.700	0.551	0.611	0.716	1.637	0.709	0.680
**C18:2*n*-5**	0.297	0.185	0.182	0.177	0.161	0.172	0.263	0.205	0.240	0.223
**C18:3*n*-3**	0.438	0.572	0.472	0.431	0.376	0.406	0.510	0.439	0.476	0.442
**C18:4*n*-3**	2.310	2.042	1.825	1.778	1.984	1.901	2.777	1.879	2.381	1.818
**C18:4*n*-1**	0.316	0.222	0.000	0.000	0.000	0.188	0.288	0.197	0.287	0.287
**C20:0**	0.240	0.288	0.217	0.000	0.107	0.206	0.228	0.258	0.203	0.261
**C20:1*n*-11**	3.010	3.420	2.946	2.859	2.380	3.639	2.838	2.418	2.353	3.027
**C20:1*n*-9**	0.113	0.150	0.000	0.000	0.000	0.144	0.124	0.138	0.119	0.140
**Δ 5.13 20:2**	0.100	0.149	0.095	0.000	0.000	0.103	0.137	0.000	0.118	0.117
**C20:4*n*-6**	1.143	1.101	0.932	0.793	0.626	1.305	0.911	0.958	0.723	1.155
**C20:4*n*-3**	0.574	0.677	0.451	0.000	0.403	0.465	0.800	0.456	0.641	0.666
**C20:5*n*-3**	20.770	19.957	19.485	19.129	22.140	19.535	22.353	19.780	22.045	20.364
**C22:1*n*-11**	3.556	4.071	3.364	3.754	3.004	5.641	2.488	2.991	4.105	3.221
**C22:4*n*-6**	0.592	0.568	0.000	0.354	0.416	0.511	0.674	0.464	0.682	0.567
**C22:5*n*-6**	0.198	0.221	0.000	0.000	0.264	0.169	0.263	0.231	0.350	0.164
**C22:5*n-3***	2.347	2.329	1.801	1.804	1.800	2.315	2.176	1.808	2.307	2.481
**C22:6*n*-3**	17.730	22.525	22.936	24.557	26.483	21.605	16.201	20.445	21.354	19.127
**∑SFA**	23.609	20.997	25.542	25.095	22.057	22.409	22.780	24.391	22.603	22.918
**∑MUFA**	24.688	24.891	22.618	22.279	20.024	25.305	25.091	23.826	21.329	25.821
**∑PUFA**	50.954	53.405	51.149	51.924	57.350	51.580	51.352	51.074	55.342	50.558
**∑*n*-3**	44.170	48.102	46.970	47.699	53.186	46.227	44.816	44.807	49.205	44.898

Supplementary Table S1 (cont.)

Fatty acid	Por1	Por2	Por3	Por4	Por5	Por6	Por7	Por8	Por9	Por10
**C12:0**										
**C14:0**	3.712	3.277	3.366	3.143	3.164	3.362	3.395	3.952	2.763	3.535
**13-methyl-tetradecanoic acid**	0.000	0.059	0.058	0.000	0.000	0.000	0.053	0.044	0.033	0.000
**C15:0**	0.467	0.388	0.383	0.419	0.314	0.411	0.345	0.396	0.341	0.397
**13-methyl-pentadecanoic acid**	0.000	0.000	0.000	0.000	0.000	0.000	0.000	0.000	0.000	0.000
**C16:0**	18.480	18.624	17.137	18.910	15.653	17.969	14.316	16.942	20.250	20.650
**C16:1*n*-7**	5.069	4.846	4.932	4.608	4.894	5.090	5.235	5.698	7.595	5.070
**15-methyl-hexadecanoic acid**	0.000	0.000	0.000	0.000	0.000	0.000	0.000	0.000	0.000	0.000
**C16:2*n*-4**	0.387	0.391	0.449	0.000	0.440	0.000	0.573	0.594	0.296	0.485
**7-methyl-6-hexadecenoic acid**	0.399	0.394	0.325	0.000	0.284	0.000	0.273	0.288	0.237	0.000
**C17:0**	0.000	0.000	0.000	0.000	0.000	0.000	0.000	0.000	0.000	0.000
**C16:3*n*-4**	0.262	0.300	0.326	0.000	0.386	0.000	0.397	0.450	0.242	0.396
**C17:1*n*-9**	0.000	0.000	0.000	0.000	0.000	0.000	0.000	0.000	0.000	0.000
**C16:4*n*-1**	0.576	0.638	0.692	0.536	0.969	0.844	0.864	1.011	0.572	0.895
**C18:0**	6.480	6.429	6.004	6.504	5.805	6.191	4.582	5.107	7.828	7.882
**C18:1*n*-9**	11.796	10.850	8.663	10.432	14.588	10.092	6.961	7.386	8.581	8.156
**C18:1*n*-6**	2.521	2.380	2.298	2.375	2.485	2.831	2.192	2.493	1.921	2.413
**C18:1*n*-5**	0.000	0.000	0.000	0.000	0.000	0.000	0.000	0.000	0.000	0.000
**C18:2*n*-6**	1.144	0.768	0.991	1.005	0.787	0.980	0.772	0.853	0.700	0.798
**C18:2*n*-5**	0.183	0.175	0.163	0.123	0.184	0.195	0.175	0.176	0.120	0.174
**C18:3*n*-3**	0.872	0.708	0.833	0.896	0.662	0.773	0.496	0.585	0.573	0.646
**C18:4*n*-3**	2.374	2.221	2.141	2.221	2.322	2.199	1.609	2.025	1.673	2.045
**C18:4*n*-1**	0.103	0.146	0.116	0.093	0.150	0.142	0.189	0.161	0.083	0.186
**C20:0**	0.441	0.250	0.409	0.371	0.465	0.423	0.336	0.343	0.604	0.476
**C20:1*n*-11**	3.567	3.130	4.069	3.046	3.547	3.515	5.811	4.901	2.998	3.239
**C20:1*n*-9**	0.145	0.092	0.114	0.342	0.124	0.000	0.168	0.000	0.000	0.000
**Δ 5.13 20:2**	0.000	0.000	0.000	0.000	0.000	0.000	0.000	0.000	0.000	0.000
**C20:4*n*-6**	0.578	0.568	0.671	0.544	0.691	0.597	0.933	0.792	0.451	0.584
**C20:4*n*-3**	0.549	0.558	0.574	0.479	0.766	0.603	0.506	0.541	0.476	0.574
**C20:5*n*-3**	14.408	14.963	16.356	15.223	19.064	16.676	15.986	17.454	13.046	16.015
**C22:1*n*-11**	2.860	3.548	6.267	2.710	1.869	3.261	11.348	6.020	2.028	4.484
**C22:4*n*-6**	0.248	0.329	0.328	0.220	0.465	0.363	0.430	0.420	0.288	0.396
**C22:5*n*-6**	0.113	0.153	0.161	0.000	0.134	0.000	0.156	0.140	0.000	0.000
**C22:5*n-3***	1.764	1.709	1.996	1.650	2.232	1.501	2.434	2.028	1.404	1.649
**C22:6*n*-3**	20.504	22.106	20.177	24.147	17.556	21.983	19.465	19.199	24.895	18.855
**∑SFA**	29.580	28.969	27.299	29.348	25.402	28.355	22.974	26.741	31.786	32.939
**∑MUFA**	25.957	24.846	26.342	23.514	27.507	24.789	31.717	26.498	23.123	23.362
**∑PUFA**	44.064	45.733	45.975	47.138	46.807	46.855	44.984	46.429	44.821	43.699
**∑*n*-3**	40.470	42.265	42.078	44.617	42.601	43.734	40.496	41.832	42.068	39.784
